# A lncRNA prognostic signature associated with immune infiltration and tumour mutation burden in breast cancer

**DOI:** 10.1111/jcmm.15762

**Published:** 2020-09-23

**Authors:** Zijian Liu, Mi Mi, Xiaoqian Li, Xin Zheng, Gang Wu, Liling Zhang

**Affiliations:** ^1^ Cancer Center Union Hospital Tongji Medical College Huazhong University of Science and Technology Wuhan China

**Keywords:** bioinformatics, breast cancer, immune infiltration, lncRNA, prognosis

## Abstract

Current studies have shown that long non‐coding RNAs (lncRNAs) may serve as prognostic biomarkers in multiple cancers. Therefore, we postulated that expression patterns of multiple lncRNAs combined into a single signature could improve clinicopathological risk stratification and prediction of overall survival rate for breast cancer patients. Two algorithms, Least Absolute Shrinkage and Selector Operation (LASSO) and Support Vector Machine‐Recursive Feature Elimination (SVM‐RFE), were used to select candidate lncRNAs. Univariate and multivariate Cox regression analyses were employed to construct a seven‐lncRNA signature for breast cancer. Stratified analysis revealed that the signature was significantly associated with multiple clinicopathological risk factors. For clinical use, we developed a nomogram model to predict overall survival and odds of death for breast cancer patients. Single‐sample gene set enrichment analysis (ssGSEA), CIBERSORT algorithm and ESTIMATE method were employed to assess the relative immune cell infiltrations of each sample. Differentially infiltration of immune cells and diverse tumour mutation burden (TMB) scores might give rise to the efficacy of lncRNA signature for predicting the overall survival of patients. Correlation analysis implied that LINC01215 was associated with multiple immune‐related signalling pathways. A seven‐lncRNA prognostic signature is a reliable tool to predict the prognosis of breast cancer patients.

## 1 INTRODUCTION

Over the last decade, with the rapid development in the depth and quality of transcriptome sequencing, long non‐coding RNAs (lncRNAs) longer than 200 nucleotides in length, which were once thought to be biological noise, were discovered in abundance. Research investigating lncRNAs has progressed notably in every field of medical research.^1^ Accumulating evidence has demonstrated that lncRNAs are involved in diverse cellular processes, including transcription initiation, chromatin modification and transcriptional regulation,^2^ by several regulatory archetypes, such as signals, decoys, guides and scaffolds,^3^ and are associated with various biological systems, such as immune, metabolic and reproductive systems, in multiple human diseases, especially cancers.^4^, ^5^ Furthermore, a large number of lncRNAs have been identified as oncogenes, such as HOTAIR and H19, which were significantly positive with poor prognosis in breast cancer,^6^ and the prognostic signatures of lncRNAs have been reported in various cancers, such as seven‐lncRNA signatures in non‐small‐cell lung cancer^7^ and six‐lncRNA signatures in glioblastoma multiform.^8^

Breast cancer (BRCA) remains a public health problem worldwide, especially for women, and the prognosis of different molecular subtypes of breast cancer patients is apparently distinct, with median overall survival for metastatic triple‐negative breast cancer being approximately 1 year compared with approximately 5 years for the other 2 subtypes.^9^ The TNM staging system developed by the American Joint Committee on Cancer (AJCC) combined with multiple molecular alteration characteristics in breast cancer patients provided a useful benchmark for establishing treatment strategies and prognostic predictions; however, these methods could not fully reflect the biological heterogeneity of breast cancer due to their diagnostic limitations and the basis of clinical information.^10^ Compared with single clinic biomarkers, integrating multiple biomarkers into a single model can improve the predictive accuracy^11^; thus, constructing novel biomarker signature associated with prognosis of efficacy of treatment seemed to be essential and effective. The construction of such gene signatures might have clinical potential to predict patient outcome and assist in treatment choice. Although there were several lncRNAs signatures published associated with breast cancer, some of them were aimed to predict the risk of recurrence^12^ or metastasis‐free survival^13^ of breast cancer patients, existing works related to prediction of prognosis of breast cancer patients were not well performed. For instance, a two‐lncRNA signature with the identification of mutated‐derived lncRNAs,^14^ an eight‐lncRNA signature based on ceRNA network^15^ and a 4‐lncRNA signature^16^ were constructed to predict survival of breast cancer patients. Nevertheless, these signatures have some certain defects regarding diagnostic limitations and accuracy of signature construction, such as lower value of AUC for ROC analysis, lacking of validation data sets or uncovering the underlying mechanism of the signatures.

In our current study, to construct a more accurate prognostic signature, we employed Univariate Cox analysis and two algorithms, LASSO and SVM‐RFE, to select significant candidate lncRNAs for further multivariate Cox regression signature construction. Then, a 7‐lncRNA signature was constituted and validated in two internal validation groups and an external validation data set GSE96058 downloaded from Gene Expression Omnibus (GEO). And stratified analysis was used to test the universal adaption of the signature in multiple breast cancer groups. Importantly, we further explored the underlying mechanism of signature from the perspective of specific characteristics of samples in different groups. Consequently, we found that our signature could divide the training and validation cohorts into high and low immune infiltration states at the immune level, and there were also significant differences in tumour mutation burden (TMB) in training cohort. Hence, we speculated that the validity of this 7‐lncRNA signature was based on the identification of patient characteristics at the immune and mutant burden levels, and such a signature would have very accurate prognostic value for clinical breast cancer patients.

## 2 MATERIAL AND METHODS

### 2.1 Data downloaded and differentially expressed analysis

Breast cancer RNA sequencing data and sample clinical information were downloaded from the TCGA database (https://tcga‐data.nci.nih.gov/tcga/), and according to the sample screening criteria (only samples owned sequencing data and clinical follow‐up information were retained), 973 breast cancers containing 150 triple‐negative breast cancer (TNBC) samples and 823 non–triple‐negative breast cancer (non‐TNBC) samples were selected as training group and randomly divided into two internal validation groups including 486 samples and 487 samples, respectively. The data process and the criteria of patients' selection were both described previously.^17^ The raw data of the training set were repurposed to the expression profiles of lncRNAs by probe reannotation based on the annotation project in the Ensembl database (http://www.ensembl.org/index.html). Expression profile matrix and patients' clinical information of external validation cohort data set GSE96058 was directly downloaded from GEO database (https://www.ncbi.nlm.nih.gov/geo/). The clinical characteristics of the patients were summarized in Table S1. In a word, the whole TCGA BRCA cohort was the training set, and subsequently, the differentially expressed lncRNAs were analysed by the R/Bioconductor package of edgeR (http://www.bioconductot.org) with the cut‐off value of |log_2_FC (fold change)| > 1 and FDR (false discovery rate) <0.01 between two subtypes of TNBC and non‐TNBC in breast cancer samples. And the differentially expressed mRNAs between the two different risk groups were also analysed by the R/Bioconductor package of edgeR with the cut‐off value of |log_2_FC| > 1 and FDR < 0.01. The differentially expressed mRNAs were visualized in a volcano plot in R.

### 2.2 Construction of 7‐lncRNA signatures of breast cancer

Univariate Cox analysis in R was used to determine the association between the expression level of differentially expressed lncRNAs and patient's overall survival (OS), and *P* < .05 was considered to be statistically significant. After filtration of differentially expressed lncRNAs, candidate prognostic lncRNAs were selected via integrated analysis of two algorithms consisting of the LASSO algorithm^18^ with penalty parameter tuning conducted by 10‐fold cross‐validation, and the SVM‐RFE algorithm searching for lambda with the smallest classification error to determine the variable.^19^, ^20^ A multivariate Cox regression model was finally used to construct a prognostic signature based on the candidate lncRNAs generated from the above filtration. A receiver operating characteristic (ROC) curve was used to estimate the accuracy and efficiency of the signature in a time‐dependent manner. All the survival analyses and graphics were conducted under the environment of R with the specific R package.

### 2.3 Implementation of single‐sample immune infiltration level analysis

The relative immune cell infiltration levels of single sample were quantified by single‐sample gene set enrichment analysis (ssGSEA) in R package gsva. The ssGSEA employed gene signatures expressed by immune cell populations to individual cancer samples.^21^, ^22^ To quantify the proportions of immune cells in the breast cancer samples, we used the CIBERSORT algorithm,^23^ which is a deconvolution algorithm that uses a set of reference gene expression values (a signature with 547 genes) considered a minimal representation for each cell type to infer cell type proportions in data from bulk tumour samples with mixed cell types using support vector regression. Using Estimation of Stromal and Immune cells in malignant tumours using Expression data (ESTIMATE) method^24^ to infer the fraction of stromal and immune cells in tumour samples, which is a specific value in order to calculate the correlation coefficient between two numerical variables.

### 2.4 Gene Ontology and pathway enrichment analysis

With the help of linear regression between the expression of mRNAs and lncRNAs, DAVID (david.ncifcrf.gov) was used to perform gene ontology analysis and Kyoto Encyclopedia of Genes and Genomes (KEGG) pathway analysis to identify the function of mRNAs in predicting the underlying biological processes of lncRNA involved in the prognostic signature. Gene Set Variation Analysis (GSVA) pathway–related analysis was conducted to explore the underlying pathway variation between two different risk groups as we have described before.^17^ The GO plot package of R software was utilized to display the results of the GO analyses, and the online website Image GP (http://www.ehbio.com/ImageGP/) was used to display the results of the KEGG analyses.

### 2.5 Availability of data and materials

Publicly available data sets were analysed in this study. The data can be found in the TCGA database: https://portal.gdc.cancer.gov/ and GEO database: https://www.ncbi.nlm.nih.gov/geo/. TCGA BRCA data set and GEO data set GSE96058 were involved in this analysis. All of those studies previously were approved by their respective institutional review boards.

## 3 RESULTS

### 3.1 Selection of candidate prognostic lncRNAs in the discovery group

**Figure 1:**
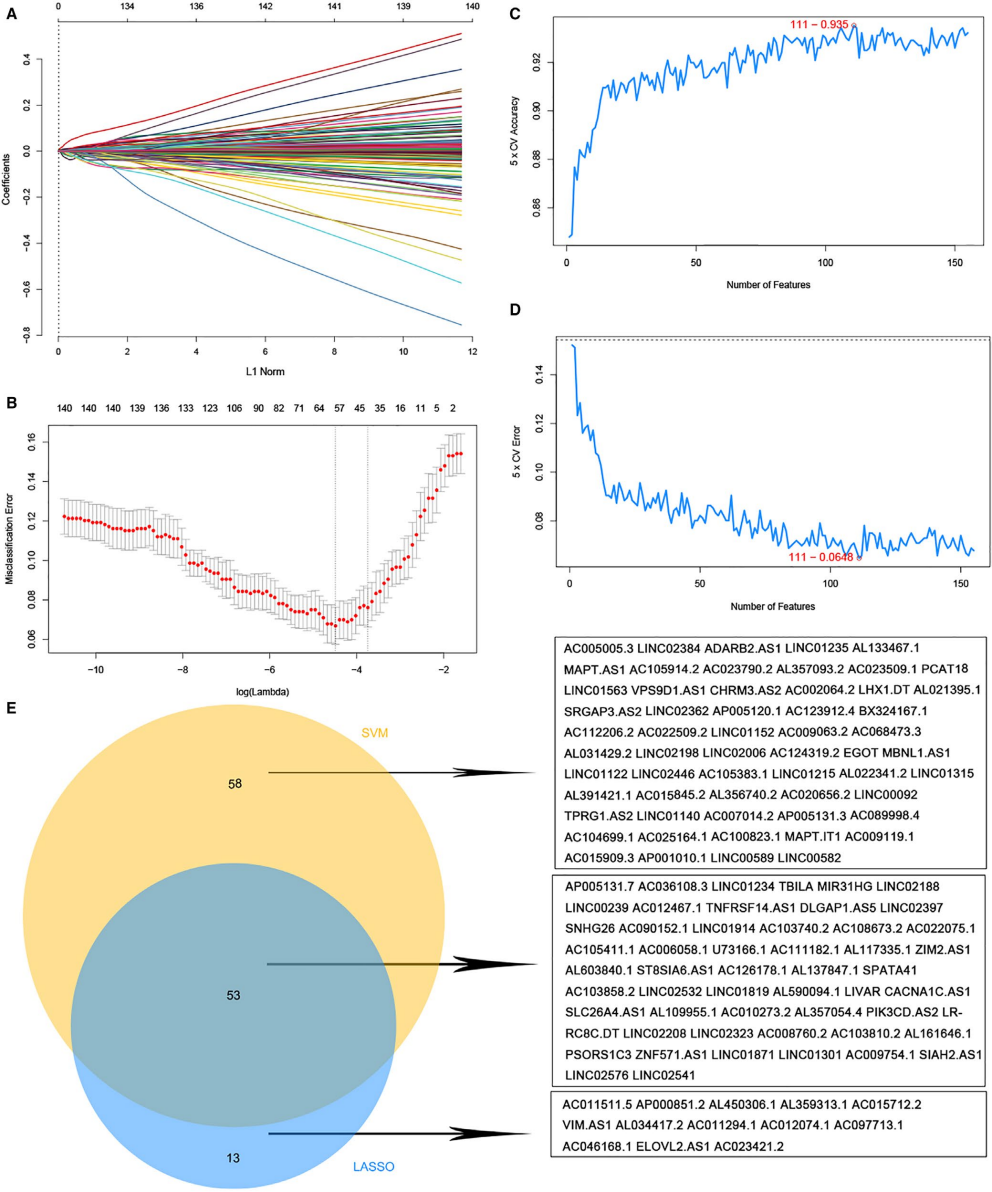
Two algorithms were used for feature selection. A, Ten‐time cross‐validation for tuning parameter selection in the LASSO model. B, LASSO coefficient profiles of 155 lncRNAs. C, The accuracy and D, the error of the estimate generation for the SVM‐RFE algorithm. E, The intersection feature selection between LASSO and SVM‐RFE algorithms and the individual components

A total of 1211 differentially expressed lncRNAs were identified between 150 TNBC and 823 non‐TNBC samples in the discovery group with the cut‐off criteria of |log_2_FC| > 1 and FDR < 0.01. Combined with survival data of these samples, 155 lncRNAs were obtained by univariate Cox proportional hazards regression analysis with *P*‐value < .05 (Figure S1). For further validation and selection of the most candidate prognostic lncRNAs with significantly characteristic value of classifying TNBC and non‐TNBC subtypes, we performed the LASSO algorithm to identify a set of 66 lncRNAs (Figure 1A,B) and the SVM‐RFE algorithm to select a set of 111 lncRNAs (Figure 1C,D). After combining the lncRNAs screened out via the LASSO and SVM‐RFE algorithms, 124 lncRNAs were identified, with 53 lncRNAs being selected simultaneously by these two algorithms (Figure 1E), which were identified as candidate characteristics of classification and prognosis.

### 3.2 Constructing a seven‐lncRNA predictive signature of breast cancer

**Figure 2:**
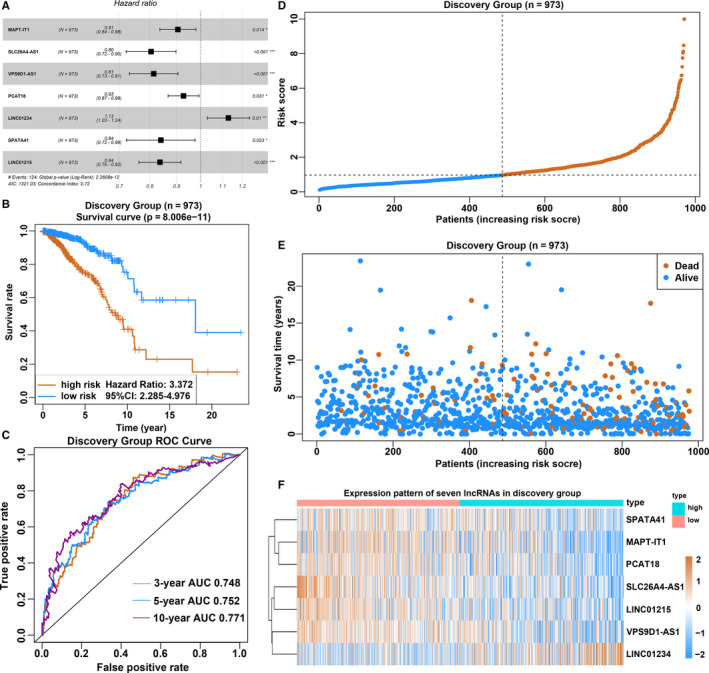
Construction of 7‐lncRNA signature. A, Hazard ratio and *P*‐value of constituents involved in multivariate Cox regression and some parameters of the lncRNA signature. B, Kaplan‐Meier survival curves were plotted to estimate the overall survival probabilities for the low‐risk versus high‐risk group in the discovery group. C, ROC curve was plotted for 3‐, 5‐ and 10‐y overall survival in the discovery group. D, The 7‐lncRNA signature risk score distribution. E, The vital status of patients in the high‐risk and low‐risk groups. F, The heatmap of the expression profiles of members in the 7‐lncRNA signature

Seven lncRNAs were identified through multivariate Cox regression analysis to construct a predictive signature in the discovery group (Figure 2A). The concordance index of this signature was 0.72 and the 95% CI = 0.66‐0.77, *P*‐value = 2.2608e−12. Using the coefficients obtained from the multivariate Cox regression, a risk score formula was constructed using the following equation: risk score = (−0.09967 * Expr MAPT‐IT1) + (−0.21712 * Expr SLC26A4‐AS1) + (−0.20558 * Expr VPS9D1‐AS1) + (−0.07476 * Expr PCAT18) + (0.121761 * Expr LINC01234) + (−0.17423 * Expr SPATA41) + (−0.17809 * Expr LINC01215). There was only one lncRNA regarded as risk factors with HR > 1, and six lncRNAs deemed to be protective factors with HR < 1 in the formula (Table 1). The prognostic score of each patient was calculated, and all 973 patients were assigned to high‐risk or low‐risk groups based on the median cut‐off point of the risk scores. The patients who had low‐risk scores were believed to have a greater chance of obtaining the same survival time than the higher risk score group (Figure 2B), and the AUC value of ROC analysis for the prognostic signature was 0.748, 0.752 and 0.771 for 3‐year survival, 5‐year survival and 10‐year survival, respectively (Figure 2C). Notably, cancer‐related death increased and the number of surviving patients decreased with increasing risk score, and every lncRNA expression value in the formula associated with the risk score is shown in the heatmap (Figure 2D‐F).

**Table 1:** Seven lncRNAs involved in the prognostic signature significantly associated with the overall survival of breast cancer patients in the discovery group

LncRNA name	Coefficient	Hazard ratio	Standard error	Z score	*P*‐value
MAPT‐IT1	−0.099668345	0.905137561	0.040347139	−2.470270472	.013501093
SLC26A4‐AS1	−0.217117229	0.804835613	0.055753203	−3.894255724	.0000985
VPS9D1‐AS1	−0.205582968	0.814172542	0.054334962	−3.783622168	.000154562
PCAT18	−0.074759138	0.927966972	0.034612703	−2.159875727	.030782291
LINC01234	0.121761038	1.129484166	0.047167774	2.581445525	.009838752
SPATA41	−0.174227615	0.840105655	0.076770442	−2.26946218	.023240235
LINC01215	−0.178089397	0.836867607	0.04680171	−3.805189941	.000141695

### 3.3 Seven‐lncRNA signature was significantly associated with OS stratified by multiple risk factors

**Figure 3:**
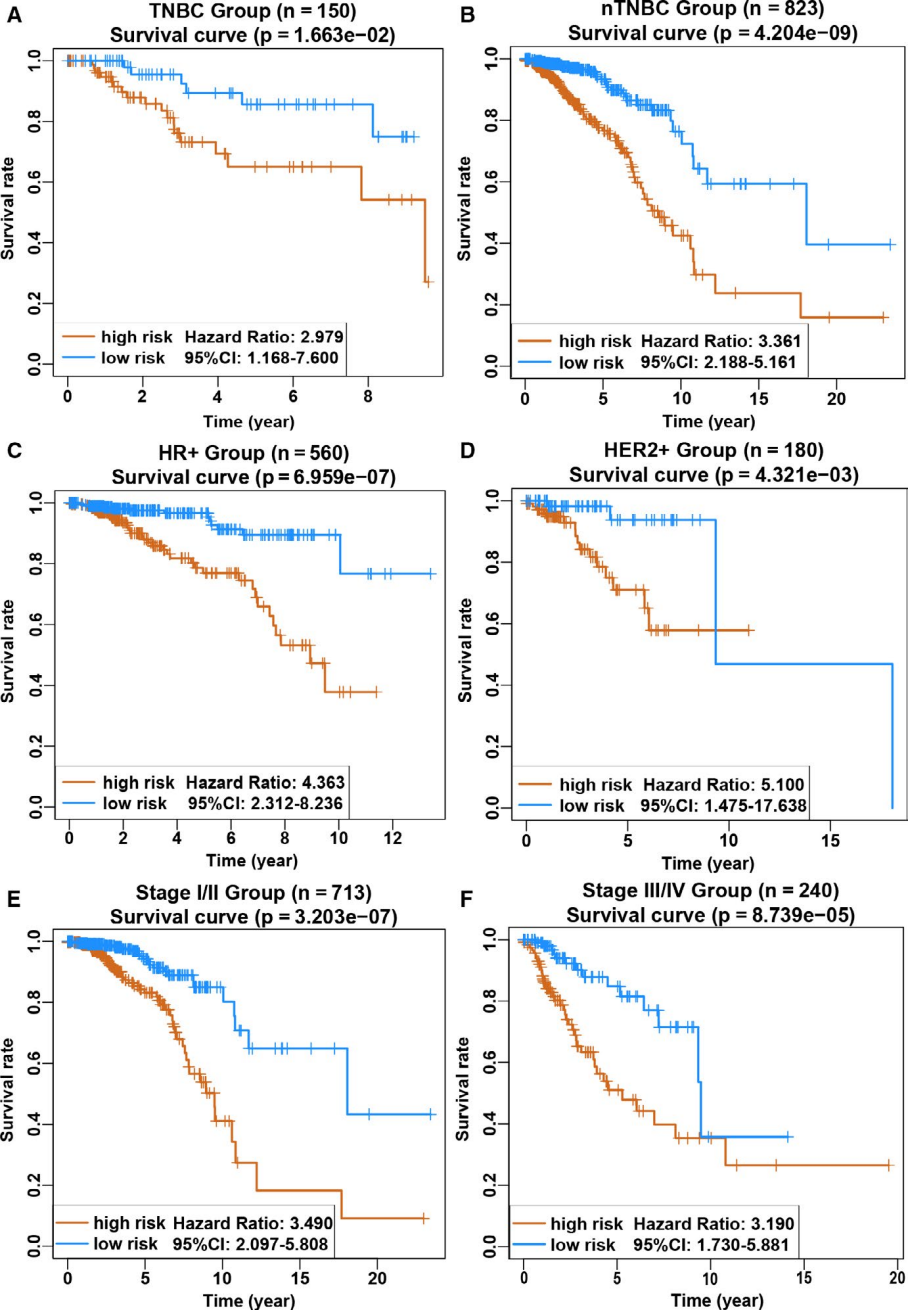
Kaplan‐Meier survival analysis for the discovery group according to the 7‐lncRNA signature stratified by clinicopathological risk factors. A‐B, TNBC and non‐TNBC groups. C‐D, Hormone receptor positive and HER2 positive. E‐F, TNM stage. We calculated the *P*‐value using the log‐rank test

**Figure 4:**
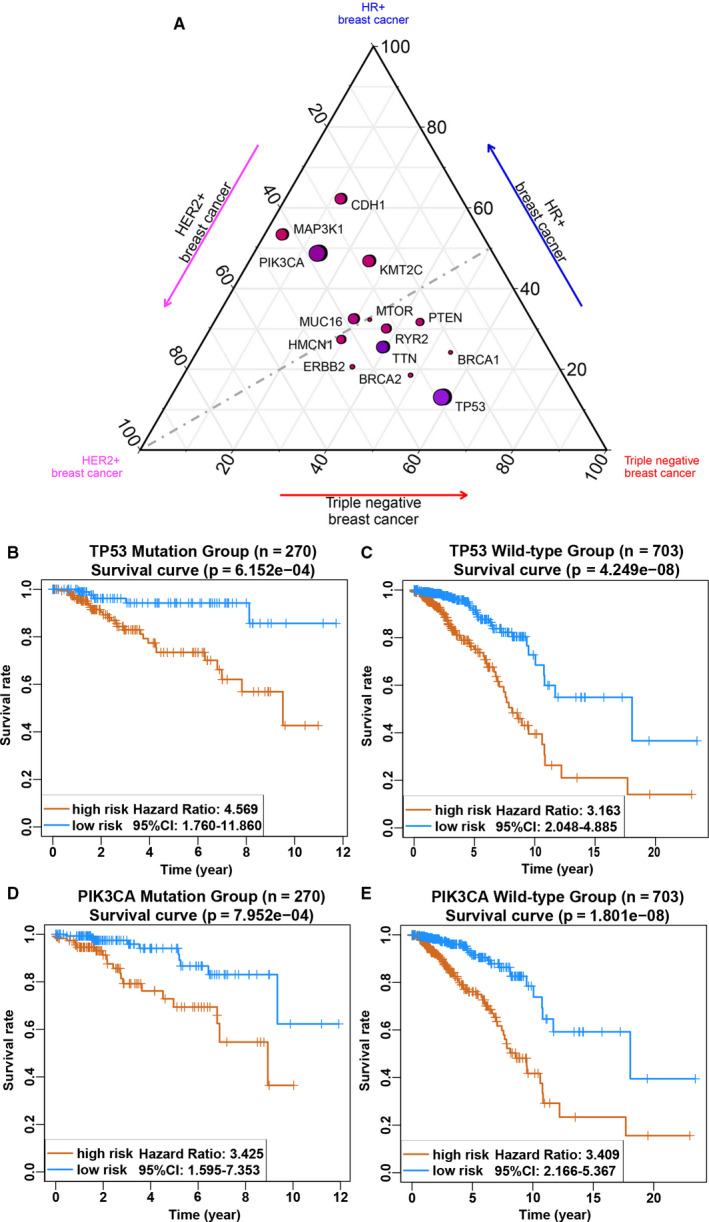
A, Ternary plot of mutation frequency in breast cancer, comparing HER2+ (left, magenta), TNBC (right, red) and HR+ (top, blue). The colour of each node indicates the relative frequency of mutations in HR+, HER2 + and TNBC, whereas the node size represents their overall frequency in all breast cancer patients. B‐C, Kaplan‐Meier estimates of the overall survival of patients carrying wild‐type or mutant TP53. D‐E, Kaplan‐Meier estimates of the overall survival of patients carrying wild‐type or mutant PIK3CA

To explore the impacts of clinical characteristics on the prognostic values of the seven‐lncRNA signature, we performed a set of predefined stratified analyses. According to the prognostic differences, the entire cohort was divided into TNBC group and non‐TNBC group, among which the latter was further separated into hormone receptor +/ERBB2‐ group and ERBB2 + group. Based on the AJCC system, patients in stages I and II were classified into the group with a good prognosis, and patients in stages III and IV were classified into the poor prognosis group. Three molecular markers, ER, PR and ERBB2, used for breast cancer typing were also used for grouping. When stratified by clinicopathological risk factors in the above groups, the seven‐lncRNA signature was still a clinically and statistically significant prognostic model (Figure 3 and Figure S2). Combined with the somatic mutation data, we found that TP53 and PI3KCA were the most frequently observed mutant genes and were associated with a higher mutation frequency in TNBC and non‐TNBC subtypes, respectively (Figure 4A). Previous studies also suggested that the mutation frequency of the above two genes might be significantly associated with poor prognosis in patients. Bearing this possibility in mind, we also implied stratified analysis based on TP53 or PI3KCA mutation status. Our data postulated that the higher risk score was associated with a higher mortality risk in the wild‐type or mutant type of these two genes in the discovery group (Figure 4B‐E). To validate the above findings, we randomly allocated the entire cohort into two internal validation groups containing 486 and 487 patients, respectively. As expected, patients in the high‐risk group had a significantly increased mortality risk compared with the low‐risk group either in internal validation group 1 and internal validation group 2 (Figure S3A‐F). Moreover, the equivalent analyses were also performed in the external validation group GSE96058 containing 3409 breast cancer samples and the risk scores of each sample were also calculated based on our lncRNA signature. Patients in the high‐risk group possessed significantly lower OS rate than those of patients in the low‐risk group (Figure S4A‐D), which was consistent with the findings from the training set, indicating that the seven‐lncRNA signature was able to accurately predict the survival of patients with breast cancer.

### 3.4 Building a predictive nomogram

**Figure 5:**
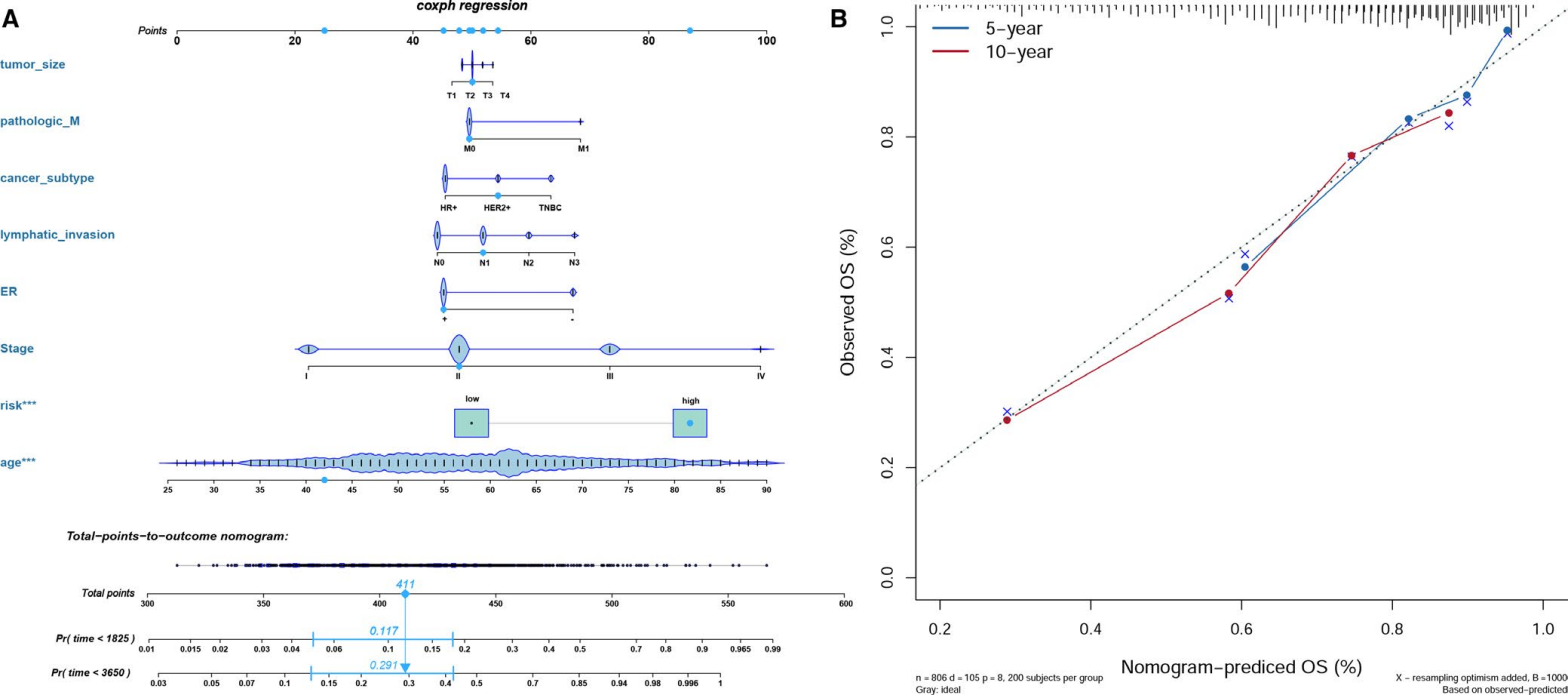
A, Nomogram to predict the 5‐y and 10‐y overall survival of breast cancer patients. B, Calibration curve for the overall survival nomogram model in the discovery group. A dashed diagonal line represents the ideal nomogram, and the blue line and red line represent the 5‐y and 10‐y observed nomograms

To develop a clinically applicable method that could predict the survival probability of a patient, we resorted a nomogram to construct a predictive model, considering clinicopathological covariates. On the basis of the univariate and multivariate analysis of OS rate (Table 2), we generated a nomogram to predict the 5‐year and 10‐year OS rates in the discovery group using the Cox regression algorithm (Figure 5A) and to predict the death odds of patients with generalized linear regression (Figure S5). The predictors included 7‐lncRNA signature, age of patients, AJCC‐T, AJCC‐N, AJCC‐M, AJCC‐stage, ER status and cancer subtype, satisfying the criteria of *P* < .05 in risk assessment. The calibration plots for the 5‐year and 10‐year OS rates were predicted well compared with an ideal model in the entire cohort (Figure 5B).

**Table 2:** Univariate and multivariate analyses of clinicopathological characteristics and 7‐lncRNA prognostic signature with overall survival in TCGA BRCA cohort

Features	Univariate analysis		Multivariate analysis	
	HR (95% CI)	*P*‐value	HR (95% CI)	*P*‐value
Age	1.539 (1.176‐2.014)	.002	1.466 (1.11‐1.936)	.007
Tumour size	1.526 (1.136‐2.051)	.005	0.839 (0.573‐1.229)	.367
Lymphatic invasion	1.727 (1.302‐2.291)	<.001	1.219 (0.857‐1.732)	.27
Pathologic metastasis	0.263 (0.176‐0.392)	<.001	0.408 (0.263‐0.634)	<.001
Tumour stage	2.189 (1.681‐2.849)	<.001	1.819 (1.226‐2.699)	.003
ER status	0.679 (0.5‐0.922)	.013	0.598 (0.436‐0.821)	.001
PR status	0.771 (0.584‐1.018)	.067	‐‐	‐‐
HER2 status	1.113 (0.777‐1.595)	.559	‐‐	‐‐
7‐lncRNA signature	2.395 (1.791‐3.203)	<.001	2.122 (1.58‐2.849)	<.001

### 3.5 Functional characteristics of the prognostic signature

**Figure 6:**
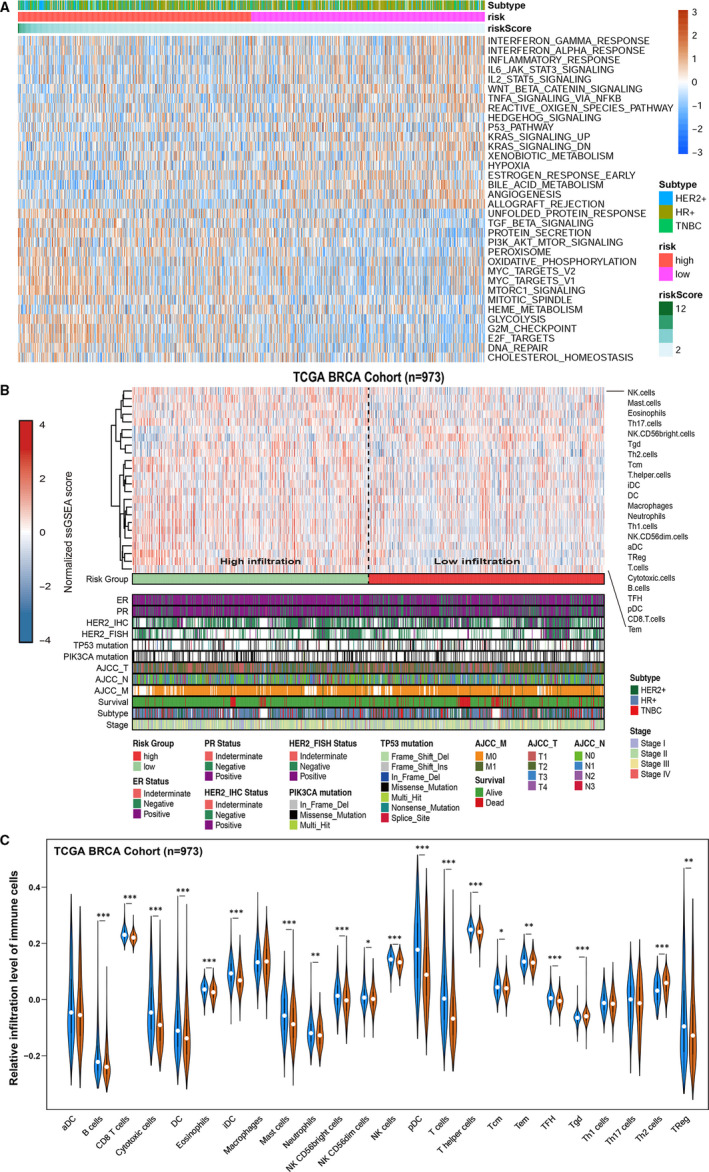
Functional characteristics of the prognostic signature. A, Differences in pathway activities scored by GSVA between high‐risk group and low‐risk group. DN, down; v1, version 1; v2, version 2. B, Heatmap of 973 patients from the TCGA BRCA cohort using ssGSEA scores from 24 immune cell types. C, Violin plot of relative infiltration level of immune cells in TCGA BRCA cohort. *P < .05; **P < .01; ***P < .001; *P* ≥ .05, not significant

To explore the underlying mechanism of the prognostic signature, again, we conducted differentially expression gene analysis between high‐ and low‐risk groups based on the lncRNA signature. After edgeR filtering (|log_2_FC| > 1 and FDR < 0.01), we screened out 595 DEGs, among which 208 genes were up‐regulated and 387 were down‐regulated in the low‐risk group compared with high‐risk group (Figure S6A,B). KEGG pathway enrichment analysis revealed that low‐risk up‐regulated genes were significantly enriched in multiple pathways, including cytokine‐cytokine receptor interaction, chemokine signalling pathway and neuroactive ligand‐receptor interaction (*P* < .05; Figure S6C). Moreover, down‐regulated genes were significantly enriched in metabolism of xenobiotics by cytochrome P450, drug metabolism‐cytochrome P450 and chemical carcinogenesis (*P* < .05; Figure S6C). Additionally, GSVA showed that patients with low‐risk scores exhibited the increased expression of proteins associated with the interferon gamma response, inflammatory response and interferon alpha response (Figure 6A). These findings indicated that there were differences in immune‐related genes and signalling pathways between high‐risk and low‐risk groups, which may partly explain the reason for the significant difference in prognosis between subgroups.

### 3.6 The risk score was associated with immune cell infiltration

The immune cell infiltration status was assessed by applying the ssGSEA approach to the transcriptomes of TCGA breast cancer cohort. Twenty‐four immune‐related terms were incorporated to assess the abundance of immune cells in tumour immune microenvironment. The whole cohort was clustered into two clusters in terms of immune infiltration by applying the lncRNA signature (Figure 6B) and the relative immune score in ssGSEA was shown in Figure 6C. Subsequently, the immune infiltration in breast cancer tissues between high‐risk and low‐risk group was investigated by the CIBERSORT algorithm. The proportion of 22 immune cells in each subgroup were shown in a bar plot (Figure S7A). The results revealed that CD8 T cells, T cell CD4 memory resting, B cell naive and B cell memory were negatively correlated with the risk score and macrophage M0 and macrophages M2 were positively correlated with the risk score (Figure S7B). For further investigating the underlying mechanism of different risk groups reflected by lncRNA signature, validation cohort GSE96058 was also calculated by ssGSEA to verify the differences in risk grouping at the immune level (Figure S7C). Correlation analysis revealed that there were similar co‐expression immune infiltration models between the training set and the validation set (Figure S7D). The population of different immune cells displayed similar expression patterns indicated that the ssGSEA algorithm was very accurate in calculating the data sets from two different sources. Interestingly, by analysing the mutation annotation files of the TCGA BRCA cohort, we found that high‐risk group owned higher tumour mutation burden score than low‐risk group (Figure S7E), which implied that poorer survival of high‐risk group may be associated with higher level of mutation.

### 3.7 LncRNA LINC01215 associated with immune‐related function

**Figure 7:**
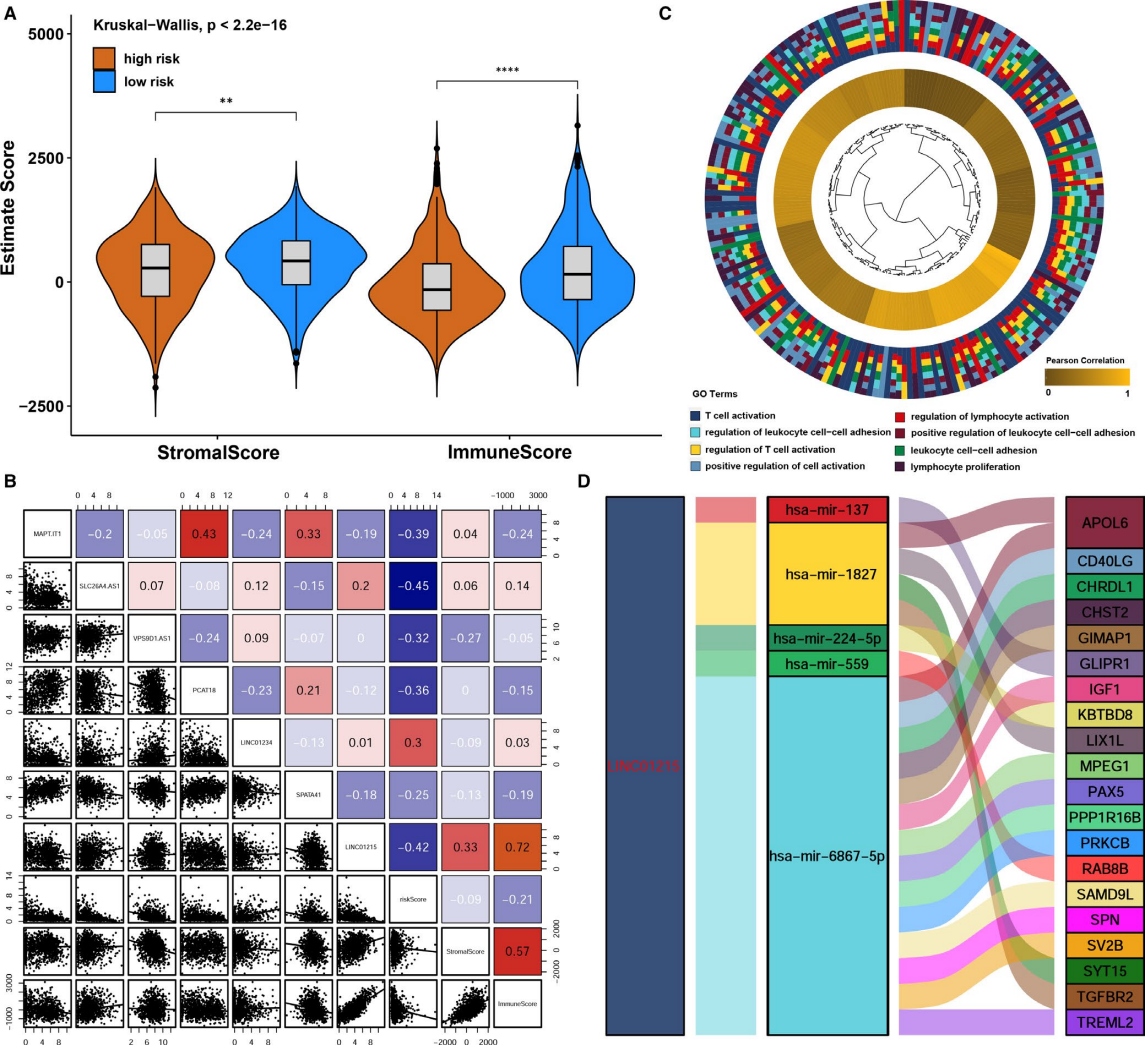
LncRNA LINC01215 function prediction. A, Stromal score and immune score were calculated via ESTIMATE method between high‐risk group and low‐risk group in TCGA BRCA cohort. B, Linear regression among members involved in lncRNA signature associated with ESTIMATE scores and risk scores, and the number in the right of the plot was coefficient. C, Go analysis of mRNAs highly co‐expressed with LINC01215. D, Sankey plot showing the ceRNA network of LINC01215

After ESTIMATE algorithm was processed, the higher estimate score was found in low‐risk group. Similarly, the fraction of immune and stromal cell was associated with low‐risk group (Figure 7A). To further elucidate the underlying biological mechanism of the lncRNAs involved in the signature, we calculated Spearman correlation coefficient among members of lncRNA signature and immune/stromal scores of ESTIMATE algorithm, only lncRNA LINC01215 was mostly positive correlated with immune scores and negative correlated with risk scores (Figure 7B). Furthermore, we used Pearson correlation analysis of the mRNAs with potential relevance to the lncRNAs in the model. We set the meaningful correlation threshold to correlation > 0.4; consequently, only lncRNA LINC01215 was predicted to associate with multiple immune‐related pathways via GO analysis among mRNAs satisfied with the cut‐off value (Figure 7C). The possibility that other components may have potential immune biological functions was not high; therefore, we regarded LINC01215 as a hub immune lncRNA in our prognostic signature. As described in our previous analysis,^25^ we set up a lncRNA related ceRNA network for LINC01215 in order to predict its possible relationships with post‐transcriptional regulation for further future (Figure 7D).

## 4 DISCUSSION

With the rapid development of bioinformatics technology, lncRNAs, which were previously considered to be transcriptional noise 1, were demonstrated by accumulating evidence to contribute to carcinogenesis and tumour progression.^26^ LncRNAs have emerged as important regulators for prognostic prediction when selecting appropriate treatment choices in a variety of human cancers, including breast cancer.^27^, ^28^ Some lncRNAs were considered to be beneficial prognostic indicators to predict prognosis in breast cancer; for instance, lncRNA GACAT3 predicted poor prognosis,^29^ and lncRNA H19 was associated with poor prognosis and promoted cancer stemness.^30^ However, due to the limited number of screened lncRNAs and unsatisfactory predictive performance, many potential and valuable lncRNAs still need to be identified to improve the predictive accuracy for breast cancer patients.^31^, ^32^ Therefore, given that the components involved in the construction of the model and the accuracy of some existing prognostic signatures were still not perfect and that the effect of the signature on different stratification groups was not well predicted, we were inclined to construct a more efficient signature of breast cancer patients.

In the present study, we found that the seven‐lncRNA signature was significantly associated with most of the stratification groups containing almost all existing clinical features of breast cancer patients. Based on the presence or absence of molecular markers for oestrogen or progesterone receptors and HER2, breast cancer was categorized into 3 major subtypes with different prognoses.^33^ The AJCC‐TNM staging system was also a useful prognostic prediction; patients with somatic co‐mutation of TP53 and PIK3CA were also associated with unfavourable survival compared with non‐carriers.^34^ Bearing these findings in mind, we conducted stratification analysis of the OS rate for patients grouping under the above conditions with the risk score obtained from the formula and, interestingly, found that the *P*‐value in all of the groups above was statistically significant. In addition, we built a nomogram to predict individual 5‐ and 10‐year overall survival rates and death odds, and the performance of the nomogram was highly consistent with the predicted model. Thus, our nomogram may provide simple, accurate prognosis predictions for breast cancer patients.

The most significant demonstration in our analysis was that we tried to figure out the underlying mechanism of different risk groups identified by our lncRNA signature. Above all, functional enrichment analysis, which indicated that risk‐related DEGs were primarily involved in multitude of immune pathways, was conducted after reclassifying the microarray according to the risk groups. We speculated that tumour immune microenvironment may has the potential to influence prognosis classification of breast cancer patients. It is worth noting that the complex interplay between tumour cells and tumour microenvironment not only plays a pivotal role during tumour development, but also has significant effects on immunotherapeutic efficacy and overall survival of patients.^35^, ^36^ Here, the immune infiltration levels of patients were assessed by three different methods and we found that patients with the better prognosis were clustered into the high immune infiltration cluster in training cohort or validation cohort. It has been reported that immune cells intratumoural and peritumoural distribution, immune cells composition and the breast tumour overall immune context and histology could influence not only the malignancy of the tumour but also the immunotherapy effect.^37^, ^38^ The high immune infiltration in the low‐risk group partly reflected the lower malignancy of the patients and the better effect of various treatments, which meant our signature could not only distinguish the survival prognosis of patients but also reflect the infiltration levels of immune cells. Moreover, the risk score was in contrast to the TMB patterns to determine the prognosis of breast cancer patients, suggesting that the poor prognosis of the high‐risk group may be due to the more mutant genes in this group. As current immunotherapy is still in its infancy for breast cancer, the patients with poor prognosis may get benefit from immunotherapy due to its high TMB score with more mutant genes.^39^

The biological function of the seven lncRNAs used in our signature has rarely been reported or studied previously. With the help of co‐expression analysis, LINC01215 was predicted to be a hub immune‐related lncRNA highly connected with multiple immune pathways, especially the T cell activation associated pathways, which was reported to be related to immune checkpoint therapy.^40^, ^41^ Combined with our correlation analysis, LINC01215 was highly positive correlated with immune score calculated by ESTIMATE algorithm and highly negative correlated with risk score, we postulated that this lncRNA took pivotal participation for lncRNA signature in distinguishing the levels of immune cells infiltration. The positive correlation between highly expressed LINC01215 and pathways highly associated with immune process suggested the importance of this lncRNA in breast cancer, meaning that such an lncRNA could serve as a potential diagnostic and therapeutic target in future research. In order to better study this promising lncRNA in the future, we set up a ceRNA network, the most common regulation form of lncRNA, to facilitate research.

In the current study, we performed a comprehensive evaluation of the prognostic signature generated and validated in our study, which is a clinically promising tool that can be used to classify breast cancer patients into subgroups with distinct outcome, immune infiltration levels and even the mutation patterns. The accuracy and universality of our model was the highest relative to previous studies.^15^, ^42^ Our current analysis should be further validated by prospective studies in multi‐centre clinical trials. Admittedly, there may be some biases in the process of selecting prognostic multi‐lncRNA signatures; nevertheless, due to this signature's high relevance to prognosis and immune infiltration, the roles of these lncRNAs merit further study, especially for breast cancer.

In conclusion, the 7‐lncRNA signature is a potential prognostic tool for predicting the overall survival rate of breast cancer patients grouped by stratification of multiple clinicopathological risk factors. A nomogram comprising a 7‐lncRNA signature may help to predict individual odds of death and help clinicians manage patients with breast cancer. Importantly, our lncRNA signature generated and validated in our study might be associated with distinct survival outcome of breast cancer patients, immune infiltration levels and even the tumour mutation burden scores.

## CONFLICT OF INTEREST

The authors declare that they have no competing interests.

## AUTHOR CONTRIBUTIONS

**Zijian Liu:** Data curation (lead); formal analysis (lead); investigation (lead); visualization (lead); writing‐original draft (lead). **Mi Mi:** Data curation (equal); formal analysis (equal); investigation (equal). **Xiaoqian Li:** Data curation (equal); formal analysis (equal); investigation (equal). **Xin Zheng:** Data curation (equal); formal analysis (equal); investigation (equal). **Gang Wu:** Conceptualization (equal); project administration (equal); writing‐review and editing (equal). **Liling Zhang:** Conceptualization (lead); funding acquisition (lead); project administration (lead); writing‐review and editing (lead).

## Supporting information

Appendix S1

## Data Availability

The data involved in this article could be downloaded directly in TCGA and GEO data sets.
