# Supplementary material for: A lncRNA prognostic signature associated with immune infiltration and tumour mutation burden in breast cancer
Source: J Cell Mol Med. 2020 Sep 23;24(21):12444–56. doi: 10.1111/jcmm.15762 (PMC7687003; doi:10.1111/jcmm.15762)
Supplement: Supplementary file 1 — Appendix S1 [file JCMM-24-12444-s001.docx]

**Supplementary material**

**FIGURE S1** Forest plot of the hazard ratio of survival-associated differentially expressed lncRNAs between TNBC and nTNBC groups.


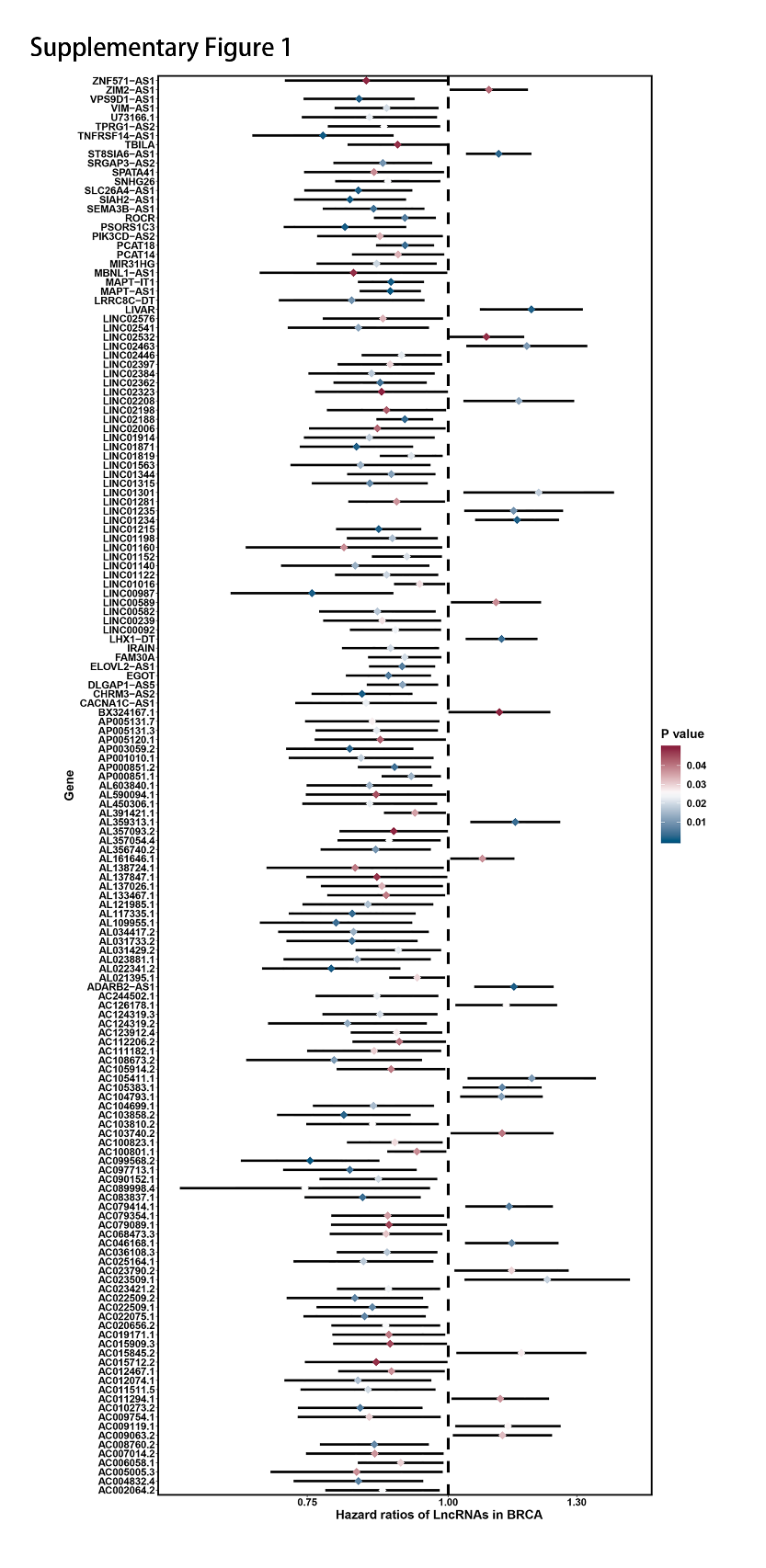


**FIGURE S2** Kaplan-Meier survival analysis for the discovery group according to the 7-lncRNA prognostic signature stratified by clinicopathological risk factors. A-B, ER+ and ER- groups. C-D, PR+ and PR- groups.


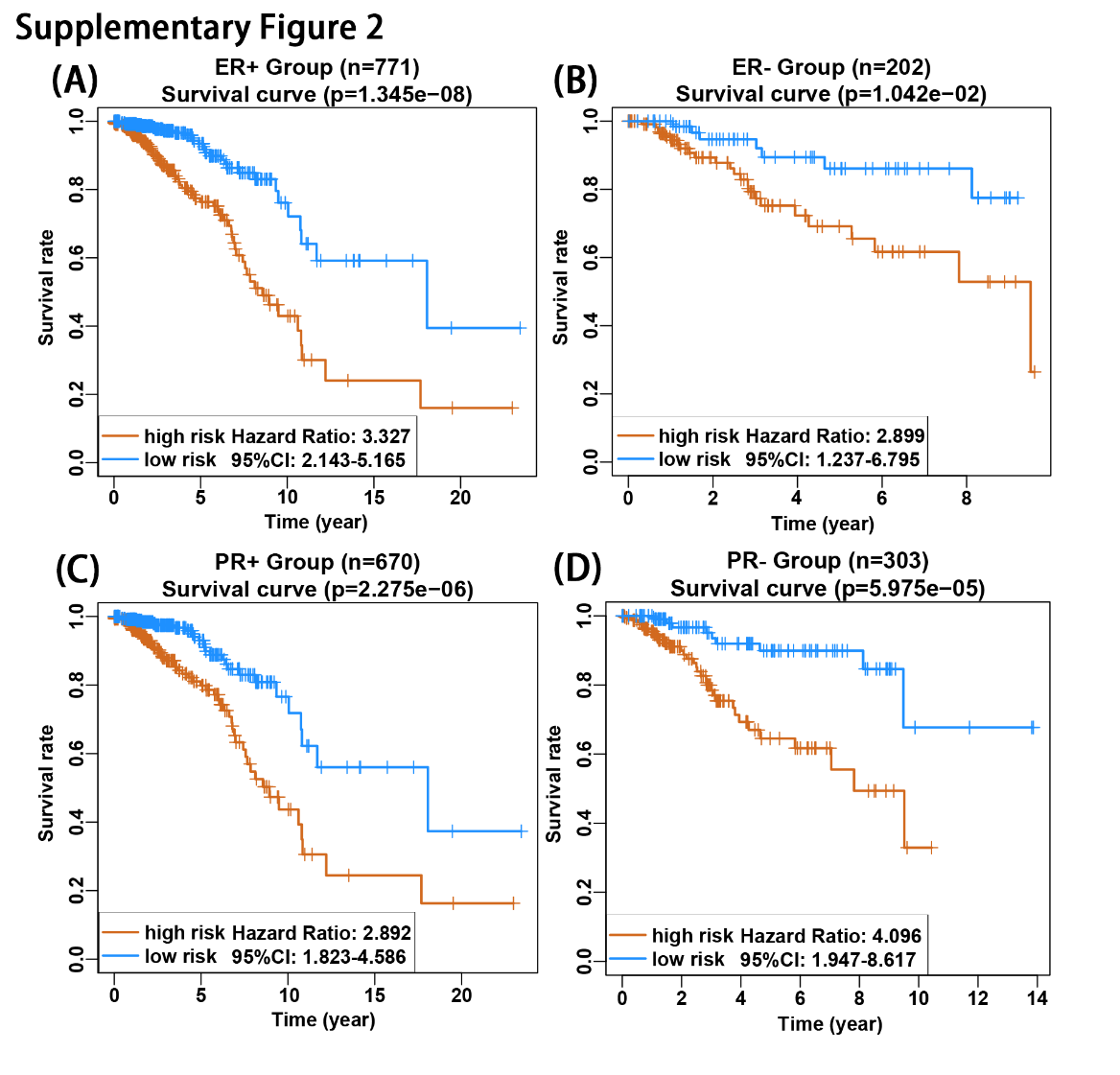


**FIGURE S3** Validation of 7-lncRNA signature. A-B, Kaplan-Meier survival curves were plotted to estimate the overall survival probabilities for the low-risk versus high-risk group in the internal validation groups 1 and 2. C-F, Survival analysis of external validation set divided into four groups containing the whole set, TNBC group, HER2+ group and HR+ group.


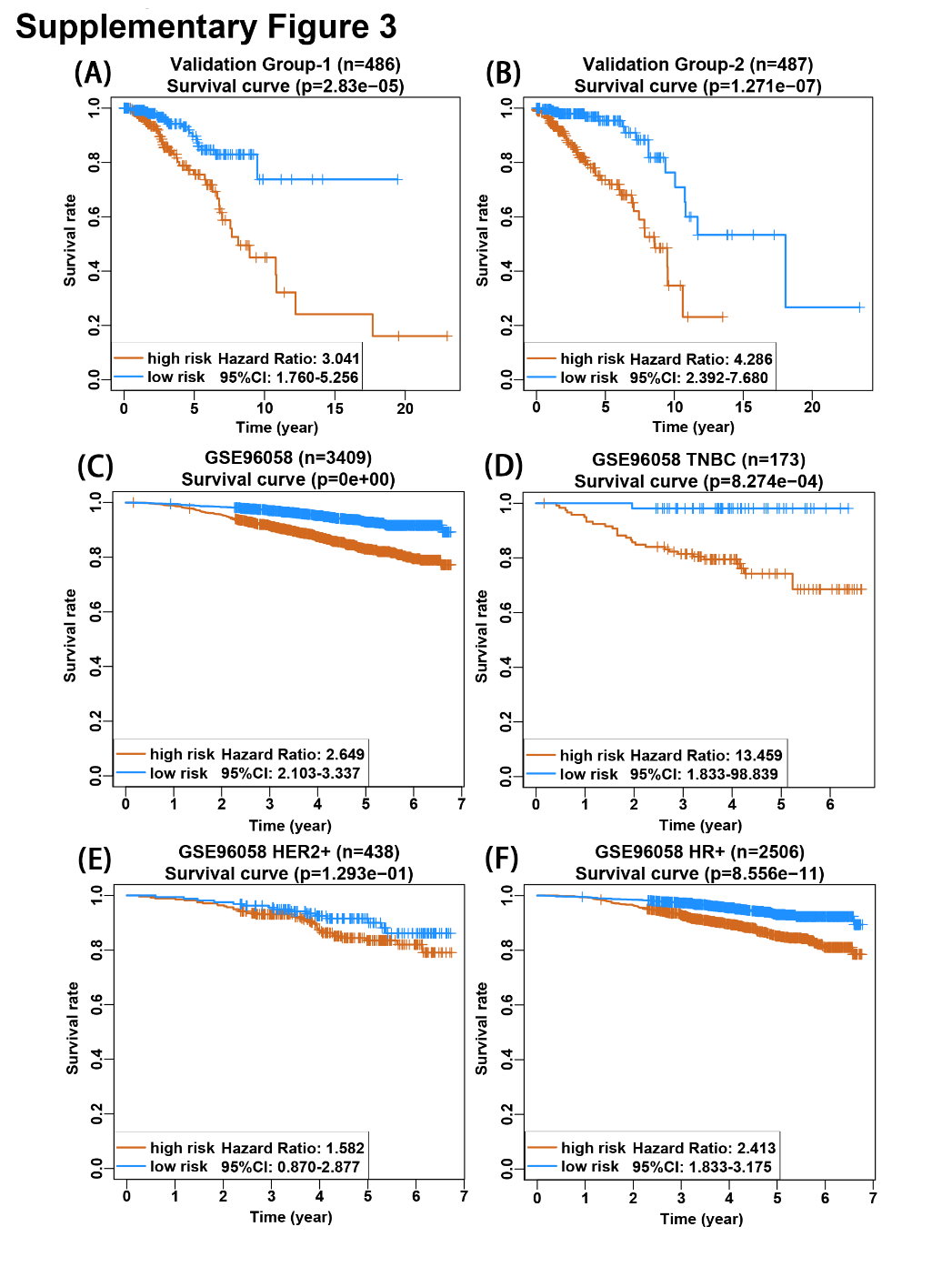


**FIGURE S4** Kaplan-Meier survival analysis for the validation group according to the 7-lncRNA prognostic signature stratified by clinicopathological risk factors. A-B, ER+ and ER- groups. C-D, PR+ and PR- groups.


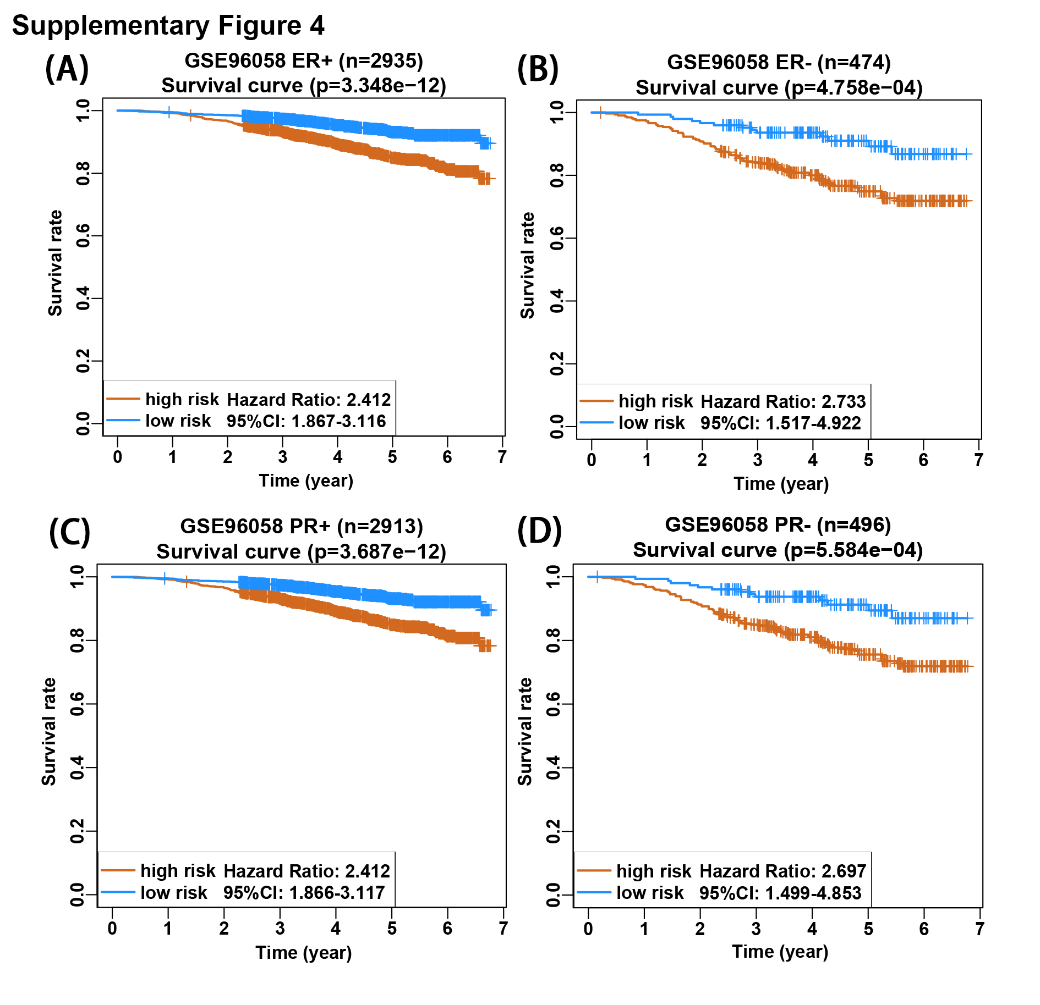


**FIGURE S5** Nomogram based on generalized linear regression to predict the odds of death for breast cancer patients.


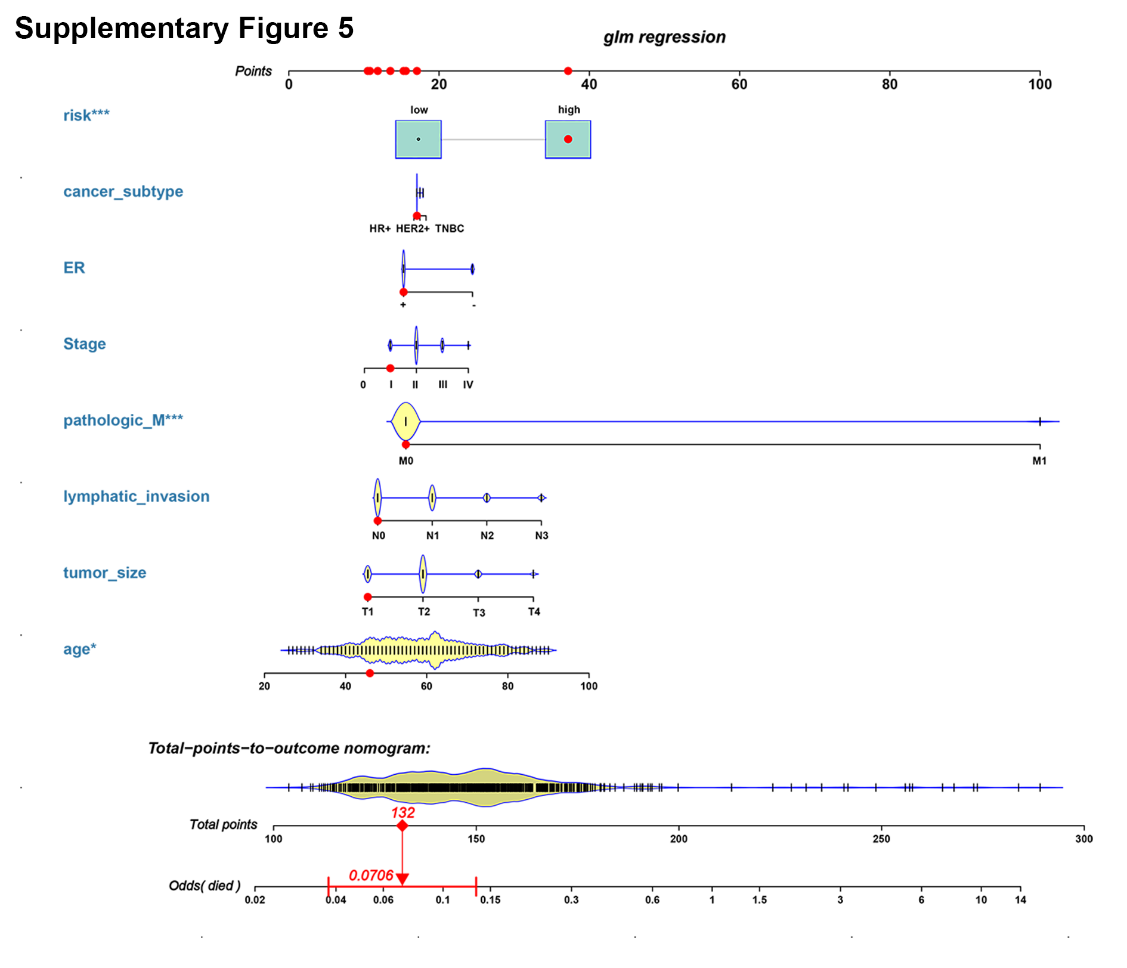


**FIGURE S6** Differentially expressed gene analysis between high risk group and low risk group. A, Heatmap of differentially expressed gene with criteria of |log_2_FC|>1 and FDR<0.01. B, The volcano plot of the differentially expressed mRNAs between high risk group and low risk group and the genes with highest fold changes were marked in the plot. C, KEGG pathway analysis about the mRNAs differentially expressed between the two different groups.


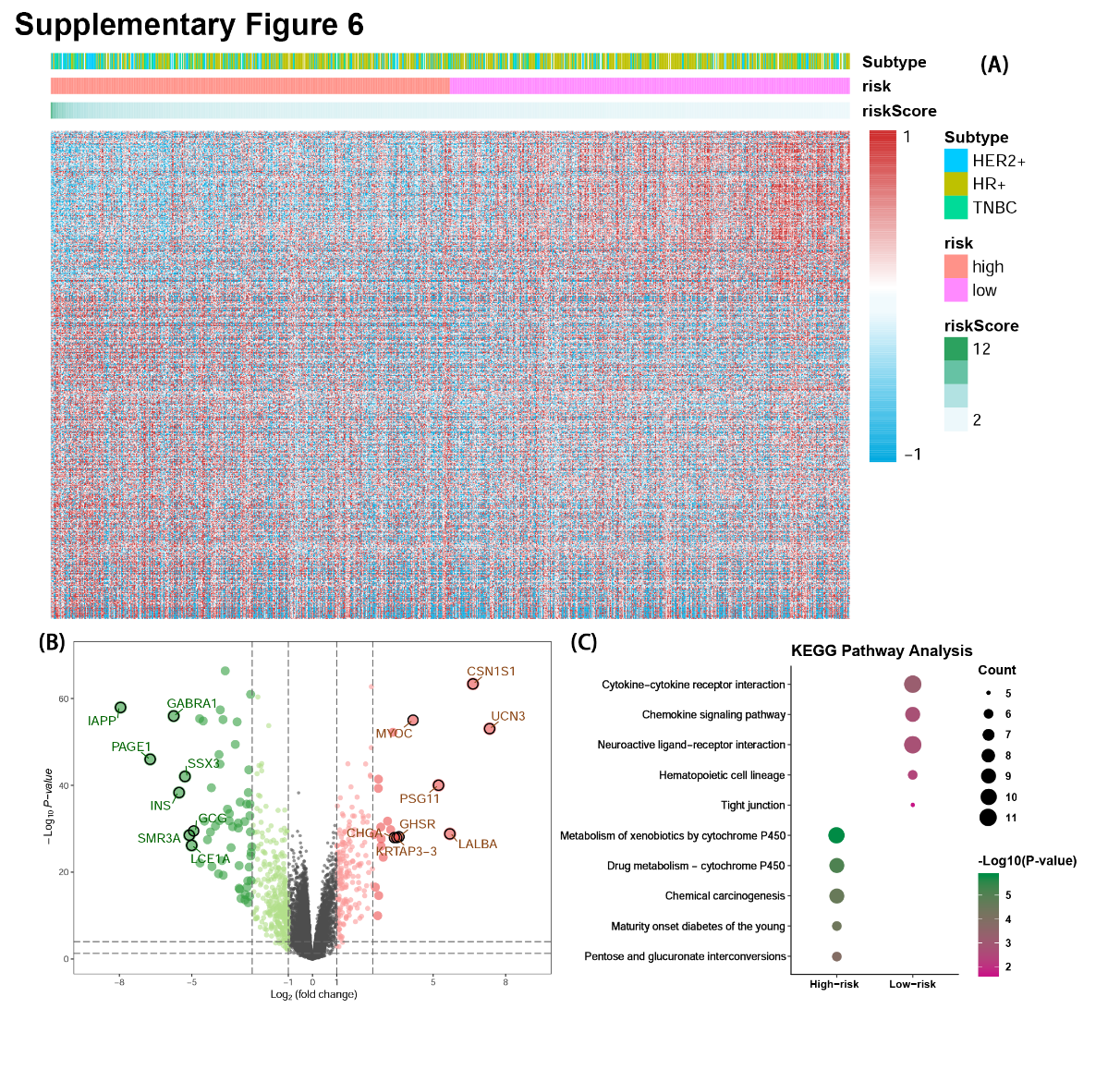


**FIGURE S7** Validation of the seven-lncRNA signature in the validation set. A, The proportion of 22 immune cells in each subgroup were shown in a bar plot. B, The heatmap showed the immune cell types that had statistical significance between the two different risk groups and the small circles for each column showed the correlation coefficient between the risk score and the relative percent of immune cells calculated by CIBERSORT method. C, The relative infiltration of immune cells calculated by ssGSEA in validation set GSE96058. *p < 0.05; **p < 0.01; ***p < 0.001; p ≥ 0.05, not significant. D, Correlation analysis among all the immune cells in the training set and the validation set. The size of the dot and the depth of the color reflected the correlation coefficient. E, TMB levels in TCGA BRCA cohort according to the risk groups.


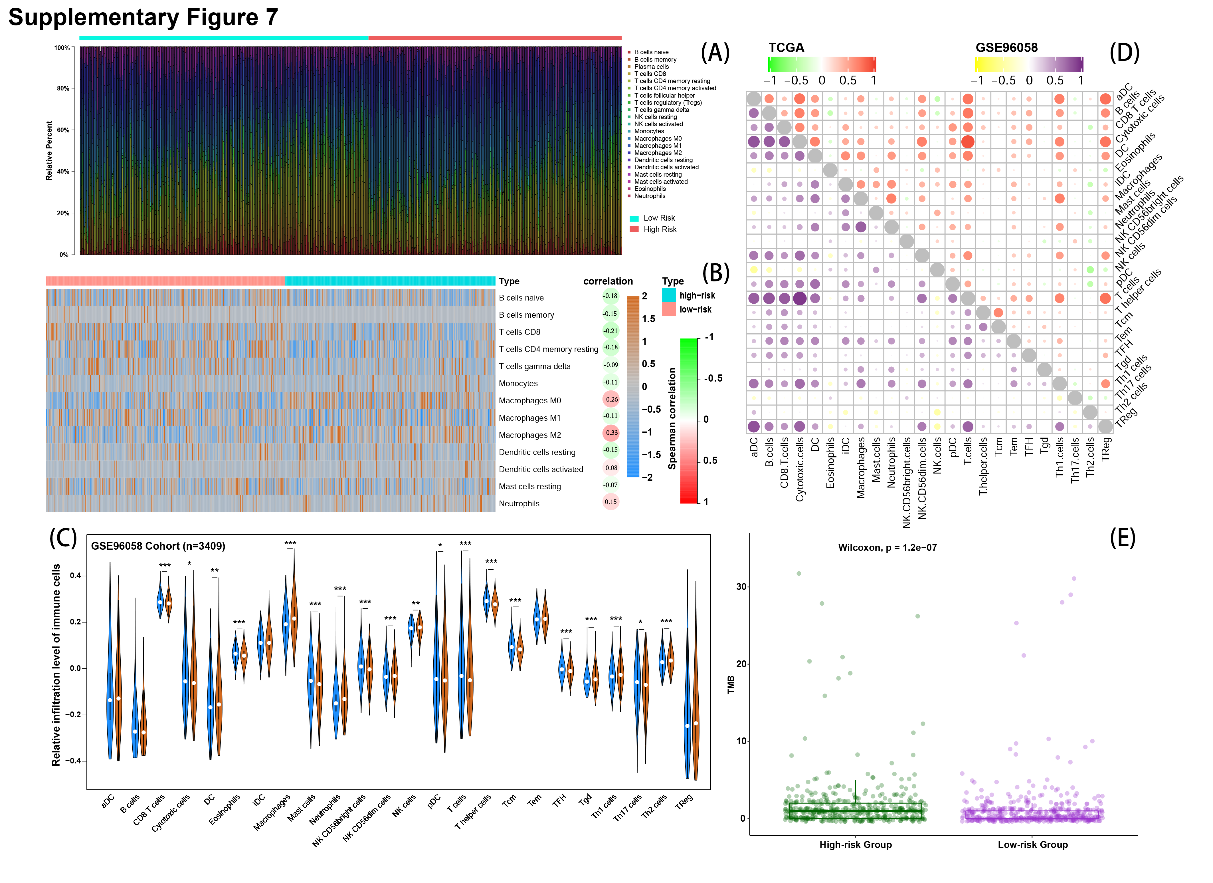


**Supplementary Table 1:** Clinical characteristics of the samples involved in our analysis.

|  | **Training cohort (n=973)** | **Validation cohort**  **(n=3409)** |
| --- | --- | --- |
| **Age, years (range)** | 58 (26,90) | 64 (24,96) |
| **Stage** |  |  |
| Stage I | 164 (16.9%) | NA |
| Stage II | 549 (56.4%) | NA |
| Stage III | 221 (22.7%) | NA |
| Stage IV | 19 (2.0%) | NA |
| **ER Status** |  |  |
| positive | 771 (79.2%) | 2935 (86.1%) |
| negative | 202 (20.8%) | 474 (14.9%) |
| **PR Status** |  |  |
| positive | 670 (68.9%) | 2913 (85.5%) |
| negative | 303 (31.1%) | 496 (14.5%) |
| **HER2 Status** |  |  |
| positive | 823 (84.6%) | 438 (12.8%) |
| negative | 150 (15.4%) | 2971 (87.2%) |
| **Subtype** |  |  |
| HR+ | 560 (57.6%) | 2506 (73.5%) |
| HER2+ | 180 (18.5%) | 438 (12.8%) |
| TNBC | 150 (15.4%) | 173 (5.1%) |
| **Vital status** |  |  |
| Alive | 849 (87.3%) | 3056 (89.6%) |
| Deceased | 124 (12.7%) | 353 (10.4%) |

ER: estrogen receptor; PR: progesterone receptor; HER2: human epidermal growth factor receptor-2; HR: hormone receptor; TNBC: triple-negative breast cancer.
